# The relevance of genetic analysis to dairy bacteria: building upon our heritage

**DOI:** 10.1186/1475-2859-3-15

**Published:** 2004-12-10

**Authors:** Christian Vadeboncoeur, Sylvain Moineau

**Affiliations:** 1 Département de biochimie et de microbiologie, Groupe de recherche en écologie buccale, Centre de référence pour virus bactériens Félix d'Hérelle, Faculté des sciences et de génie, Faculté de médecine dentaire, Université Laval, Quebec City, Quebec, G1K 7P4, Canada

## Abstract

Lactic acid bacteria (LAB) are essential for the manufacture of fermented dairy products. Studies on the physiology, biochemistry and genetics of these microorganisms over the last century have contributed considerably to the improvement of fermentation processes and have resulted in better and safer products. Nevertheless, the potential of LAB is far from being maximized. The sophistication of biotechnologies and the availability of complete genome sequences have opened the door to the metabolic engineering of LAB. In this regard, the recent publication of the complete genome sequences of two *Streptococcus thermophilus *strains will provide a key tool to facilitate the genetic manipulation of this important dairy species.

## Introduction

The souring of milk by microorganisms has been used for thousand of years as a natural preservation procedure [1]. The method was domesticated to manufacture man-made edible fermented products such as cheese, which appeared in the human diet some 8,000–10,000 years ago. However, up to the beginning of the 20th century, milk fermentation was by no means a controlled process, and the search for improvements was strictly empiric and based on trial and error.

The discovery and characterization of lactic acid bacteria (LAB) has tremendously modified the way fermented milk products are made. Considerable efforts have been devoted over the last fifty years to increase our knowledge about the genetics, biochemistry, and physiology of LAB. In addition to enhancing our understanding of microbial life, these studies have allowed dairy microbiologists and cheesemakers to select better strains and improve the productivity, quality, and safety of the final products. The characterization of LAB has promoted the rational development of blends of defined bacterial strains, now known as starter cultures, which are increasingly replacing the undefined blends traditionally used by the dairy industry. This strategy makes it easier to control acid production and, to some extent, phage problems. Unfortunately, it is also widely perceived that this approach reduces the much sought-after rich, complex flavour of fermented products made with undefined cultures. Thus, the search for LAB strains that produce characteristic aroma and flavours remains an attractive challenge for the dairy industry.

The increasing awareness of the population of the importance of a healthy diet has led scientists to revisit the century-old hypothesis that some specific fermented dairy products may provide health benefits. A plethora of studies are underway to evaluate this concept and to improve the health benefits of LAB. Also, the long history of the safe use of these bacteria has made them very attractive candidates as vaccine delivery vehicles [2,3]. Lastly, thanks to their relatively small genome and simple metabolism, LAB can be exploited as food-grade cell factories to produce molecules of industrial and therapeutic interest.

To meet some of these challenges and promises, it became apparent that metabolic engineering was the best approach. Owing to the industrial interest, the sound knowledge of its genetic and the availability of genetic tools, metabolic engineering of LAB has been carried out mainly with *Lactococcus lactis*. The publication of the complete genome sequence of *L. lactis *in 2001 [4] was a boost to speed up achievements in rerouting metabolic fluxes with this LAB. For instances, it became possible to increase the production of diacetyl and to transform this mesophilic bacterium from homolactic fermenter to homoalanine fermenter. The innovative production of vitamins by *L. lactis *via genetic manipulation [5,6] is a convincing example of how metabolic engineering of LAB can be beneficial for consumers. A number of reviews [7-12] provide a more comprehensive discussion of the metabolic engineering of *L. lactis*.

Recently, the complete genome sequence of two *Streptococcus thermophilus *strains were published [13] and a third one is nearing completion [14]. *S. thermophilus *is a thermophilic LAB species widely used for the manufacture of yogurt and cheeses that require elevated cooking temperatures such as Swiss and Italian types. The availability of these genome sequences will make it possible to apply genomic-related analytic methods such as functional and comparative genomics, microarray technologies, proteomics, and bioinformatics to this species. These genomic-based technologies will undoubtedly accelerate the metabolic engineering of *S. thermophilus*, which is still at its infancy. We will briefly discuss current studies that will greatly benefit from these *S. thermophilus *genomic sequences: food-grade vectors, optimization of lactose metabolism and exopolysaccharide (EPS) production, and resistance to bacteriophages.

## Discussion

### Food-grade vectors

Metabolic engineering often involves inactivation or overexpression of relevant chromosomal genes or stringent control of an extrachromosomal foreign gene borne by a plasmid artificially introduced into the host. Plasmids thus play a central role in these studies because they are the primary vehicles used to manipulate target DNA sequences. However, these extrachromosomal genetic elements are naturally but infrequently observed in *S. thermophilus***** strains[15].

Understandably, very few cloning tools are at hand, and those that are available are based on similar replication machinery. This lack of diversity poses significant problems for many genetic studies. While several types of transformation methods have been developed to introduce foreign DNA into *S. thermophilus*, an efficient, stable, food grade expression system is still lacking. The recent discovery of a novel theta-type replicating plasmid [15] as well as suitable selection markers effective in *S. thermophilus*, such as galactokinase [16] and alpha-galactosidase [17], suggest that help is on the way. The complete *S. thermophilus *genome sequences also provide crucial information on transcription and translation initiation signals and the codon usage of this bacterium. This may help in the design of efficient expression systems.

### Lactose metabolism

The ability of *S. thermophilus *to rapidly take up and metabolize lactose is crucial in several fermentation processes. While *S. thermophilus *readily metabolizes the glucose moiety of lactose, it is unable to metabolize galactose, which is expelled into the external medium. The presence of galactose in yogurt and other dairy products might be unwanted for different reasons. Notably, galactose is poorly metabolized by humans and may cause, under certain conditions, health problems [18,19]. The release of galactose by *S. thermophilus *results either from poor expression of the *gal *operon [20] or inefficient translation of the *galK *gene coding for galactokinase [16,21]. The finding that the inability to grow on galactose was a consequence of poor *galK *translation was first suggested by a comparative analysis of the *S. thermophilus gal*-*lac *operons with the homologous genes from the phylogenetically related oral bacterium *Streptococcus salivarius*. This is a convincing example of how comparative genome analysis may help to decipher specific metabolic pathways of LAB. While the introduction of a functional extrachromosomal *S. salivarius galK *allele in *S. thermophilus *allows it to grow on galactose, it does not prevent the expulsion of galactose during growth on lactose [16]. In this context, it is noteworthy that 10% of *S. thermophilus *genes are not functional (pseudogenes) and that one third of these pseudogenes are dedicated to sugar metabolism [13]. It would be interesting to determine whether reactivation of one or several pseudogenes that were originally involved in sugar metabolism could prevent or decrease galactose expulsion during growth on lactose. In this context, the availability of the complete sequence of the *S. salivarius *genome would be welcome since many *S. thermophilus *pseudogenes involved in sugar transport are functional in *S. salivarius *[13].

### Exopolysacharides

Some strains of *S. thermophilus *are widely used for the commercial manufacture of yogurt because they produce exopolysaccharides (EPS), which give a viscous texture to the fermented dairy product. Other EPS-producing *S. thermophilus *strains can also enhance the functional properties of some cheeses such as Mozzarella. The organoleptic properties of these products are largely due to the amount and types of exopolysaccharide produced during the fermentation process. The yield and sugar constituents of EPS are influenced by several factors and vary from strain to strain [22]. *S. thermophilus *does not naturally produce large amounts of EPS, which explains why considerable efforts are being directed toward understanding the cellular mechanisms of EPS biosynthesis. Since low levels of sugar precursors may limit EPS synthesis, a better understanding of *S. thermophilus *metabolic flux during sugar metabolism may lead to new strategies to enhance EPS production [22]. Levander et al. [23] showed that such knowledge can be used to enhance EPS production in *S. thermophilus *by metabolic engineering of central carbon metabolism. Moreover, because EPS yields are growth associated, efforts to increase production levels are likely to require novel strategies to enhance biomass production. This task clearly requires a comprehensive view of the cell machinery. The sequencing of a whole genome is a mandatory step to achieve this goal. As our knowledge of *S. thermophilus *continues to improve, novel EPS and/or applications for EPS^+ ^cultures are likely to emerge.

### Phage resistance

Bacteriophages are the most significant cause of fermentation failures in the dairy industry worldwide. Dairy microbiologists have attempted for more than 70 years to eliminate, or at least bring under better control, the bacteriophages that interfere with the manufacture of many fermented milk products [24]. The publication of the complete genomes of two *S. thermophilus *hosts should provide new insights in several areas of phage research.

The development of bacteriophage-insensitive *S. thermophilus *mutants is generally the first approach used to transform a phage-sensitive strain into a phage-resistant mutant, most likely following spontaneous chromosomal mutation in the gene encoding the phage receptor. Although progress has been made in identifying phage proteins involved in *S. thermophilus *host recognition [25], the identification of the phage receptors on the cell surface has remained elusive. Based on the results of genome data mining [26], a number of potential receptors can now be experimentally verified.

At least eight complete *S. thermophilus *phage genomes are now available [27,28]. Phage research has thus already entered into the post-genomic era. Microarrays covering the two main groups of *S. thermophilus *phages are already available[29], meaning that it is now possible to design a complete array containing host and phage genes to study phage-host interactions on a novel and global scale during the infection process.

## Conclusions

A thorough understanding of LAB metabolism and how it is regulated by external stimuli is a prerequisite for maximizing the potential of LAB. The availability of complete *S. thermophilus *genome sequences will obviously facilitate our understanding of the metabolic potential of *S. thermophilus*. It will also make it easier to design rational genetic manipulations of this important dairy bacterium in order to produce added value cheeses and yogurts and to use it as a cell factory. In addition, knowing the complete genome sequences should lead to the development of new genetic tools that will provide insights into the evolution of microbial communities, shifts in metabolism, and how each member adapts to the environmental changes that occur during complex fermentation processes.

The exciting biotechnological developments in LAB and studies that are already underway will benefit both consumers and the dairy industry. The availability of the complete *S. thermophilus *genome sequence have opened up exciting, new possibilities that will build on an already rich heritage.
